# Supplementary material for: Substrate-engaged type III secretion system structures reveal gating mechanism for unfolded protein translocation
Source: Nat Commun. 2021 Mar 9;12:1546. doi: 10.1038/s41467-021-21143-1 (PMC7943601; doi:10.1038/s41467-021-21143-1)
Supplement: Supplementary file 1 — Supplementary Information [file 41467_2021_21143_MOESM1_ESM.pdf]

Supplementary Information for:

Substrate-engaged type III secretion system structures reveal gating mechanism for unfolded protein translocation

Sean Miletic, Dirk Fahrenkamp, Nikolaus Goessweiner-Mohr, Jiri Wald, Maurice Pantel, Oliver Vesper, Vadim Kotov, and Thomas C. Marlovits

This document contains:

Supplementary Figures 1 – 26

Supplementary Tables 1 – 4

Supplementary References

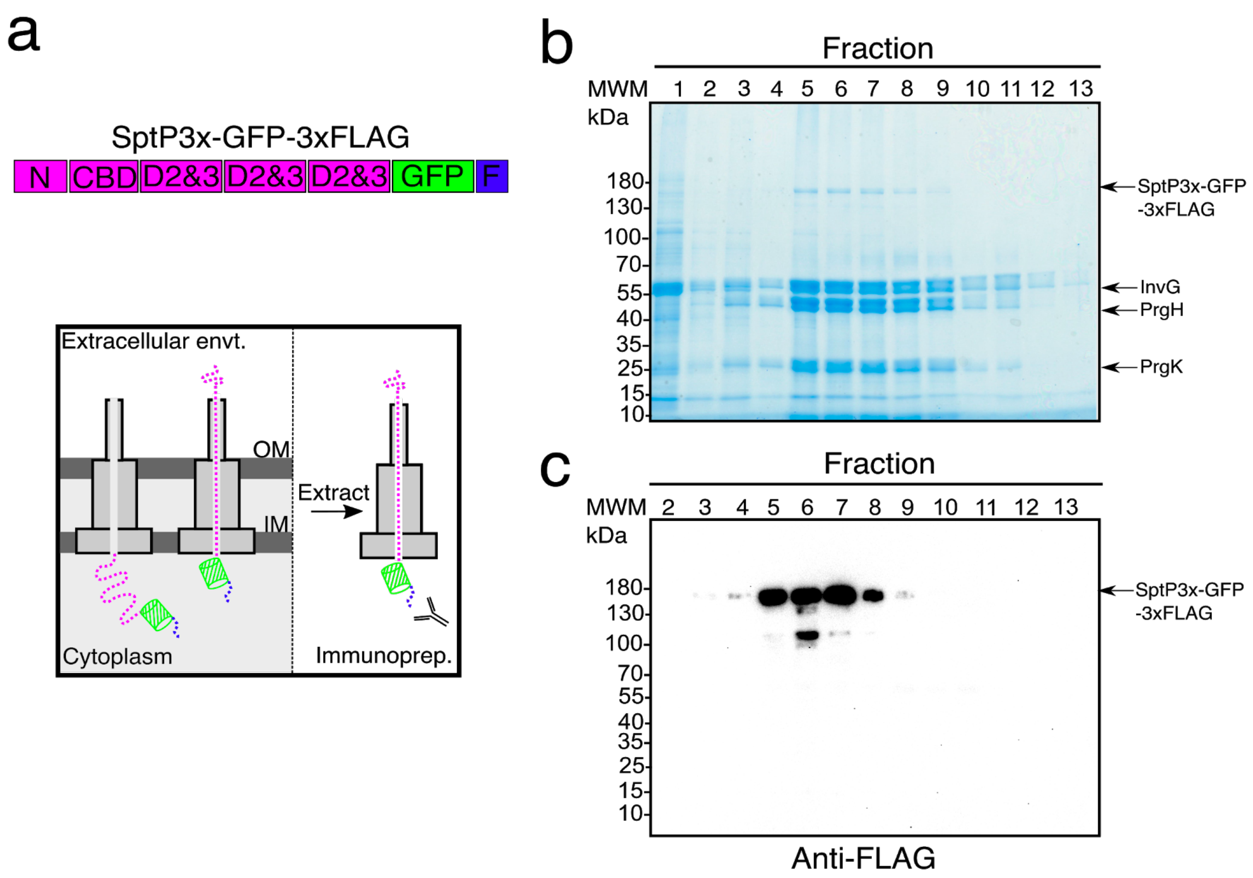

Supplementary Figure 1: Needle complex purification. **a**, Domain organization of the engineered SptP construct used in this study. N: N-terminal signal sequence; CBD: SicP-specific chaperone binding domain; D2&3: repeats of SptP effector domains 2 and 3; GFP: Unfolding resistant enhanced green fluorescent protein; F: 3xFLAG protein purification tag for pulldown experiments. Below panel: schematic on trapping substrates in needle complexes and subsequent immunoprecipitation. OM: outer membrane, IM: inner membrane. Substrate shown in magenta with a green cartoon representing GFP, and blue 3XFLAG tag. **b**, Coomassie-blue stain of CsCl gradient fractions obtained during needle complex purification. SptP3x-GFP migrates to its approximate molecular weight of 176 kDa. **c**, Western blot analysis of CsCl gradient fractions in (**b**) probed with an anti-FLAG antibody. Images in (b-c) represent one experiment of several successful repetitions of needle complex purifications.

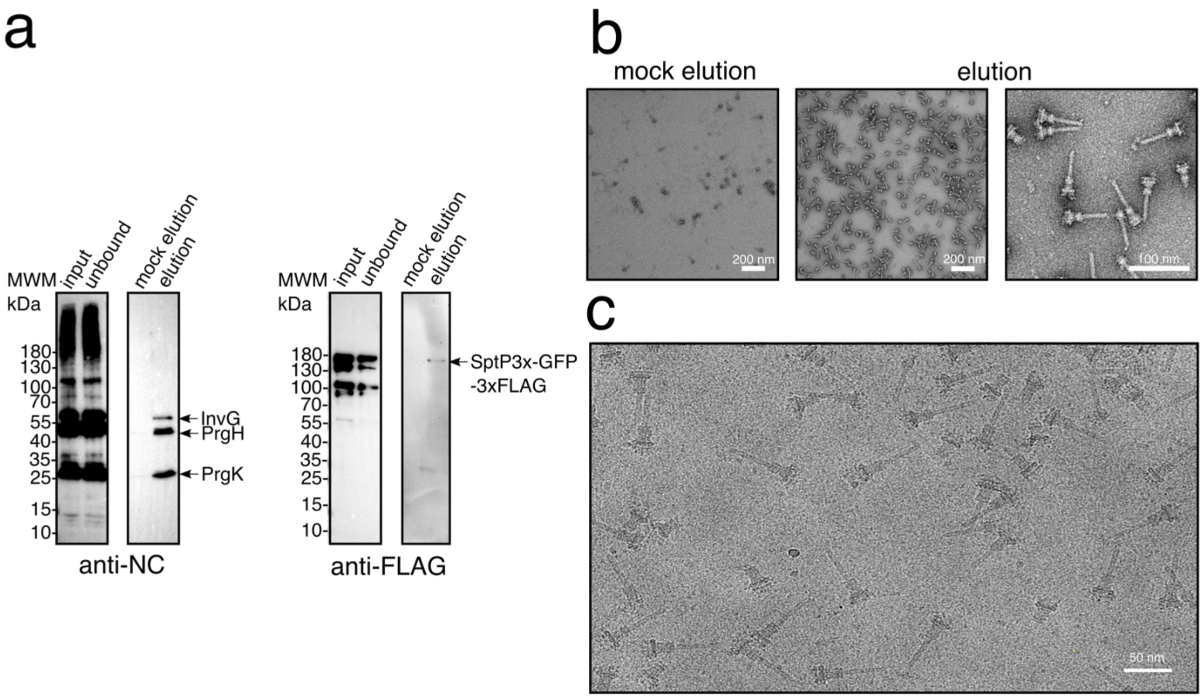

Supplementary Figure 2: Immunoprecipitation of substrate-engaged needle complexes. a, Western blot analysis of needle complex pulldown experiments using anti-FLAG magnetic beads probed with anti-needle complex antibodies recognizing inner (PrgK/H) and outer ring (InvG) proteins (left) and anti-FLAG antibody (right). Unbound: Supernatant of pulldown sample. Mock elution: Elution step with buffer lacking the FLAG peptide eluent. **b**, Negative-stain TEM of samples taken from mock (left) and FLAG peptide (right) elutions at two magnification levels. Scale bars: 100 nm and 200 nm. **c**, Representative micrograph of plunge frozen substrate-engaged needle complexes. Scale bar: 50 nm. The blots and micrographs shown here represent one of three successful pulldown experiments.

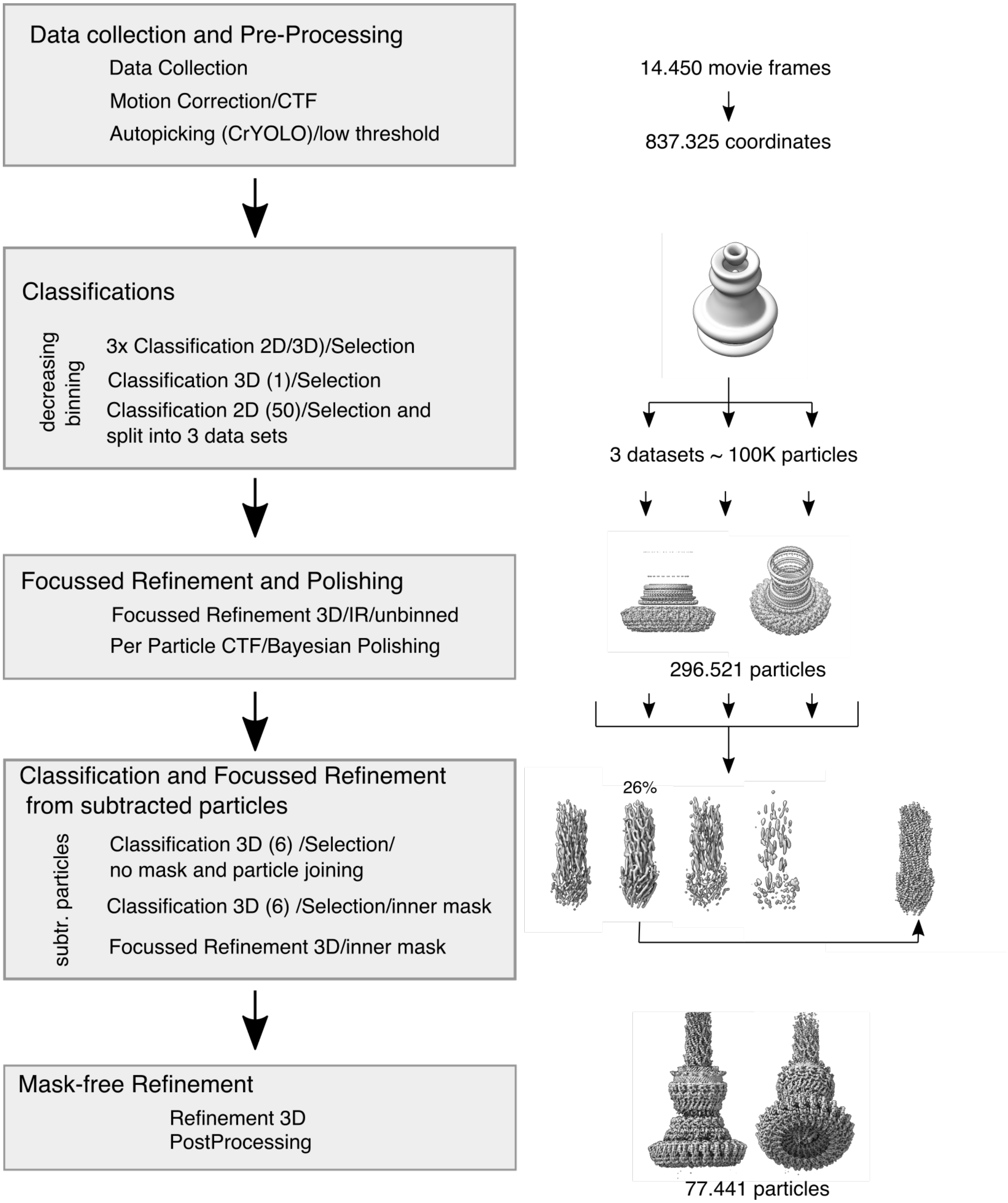

Supplementary Figure 3: Cryo-EM data collection and single particle reconstruction procedure.

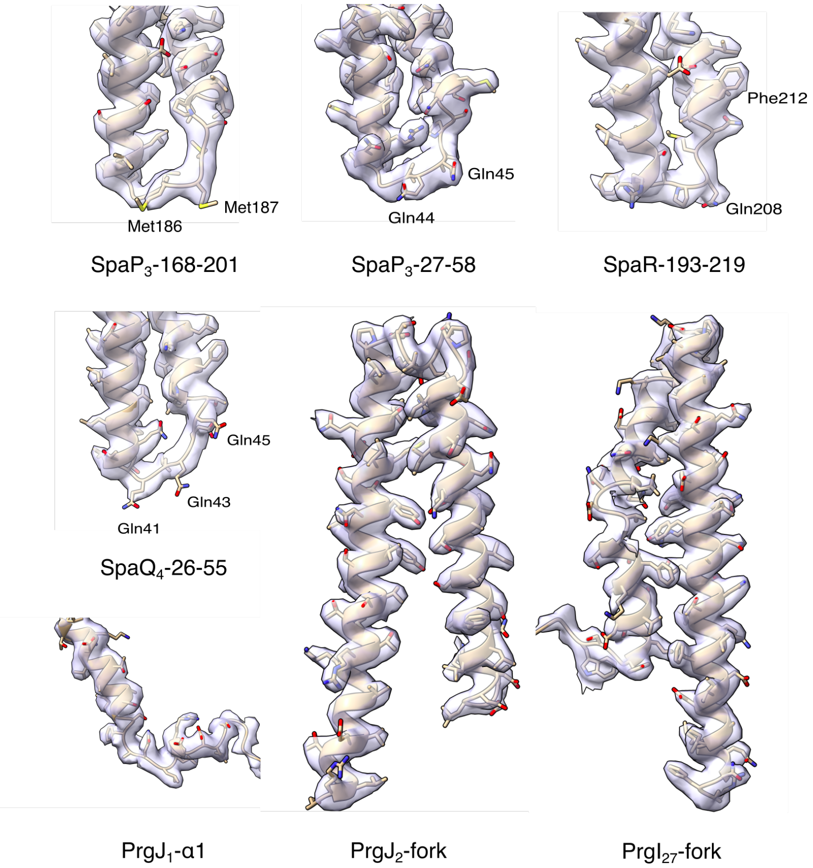

Supplementary Figure 4: Example cryo-EM densities. Shown are regions containing residues directly involved in substrate translocation through the EA and protein components building the inner rod (atrium) and filament (tunnel). Met186 and Met187 of $SpaP_3$ (M-gate), Gln44 and Gln45 of $SpaP_3$ (Q2-belt), Gln208 and Phe212 of SpaR (Q1-belt/M-gate) and Gln41, Gln43 and Gln45 of $SpaQ_4$ (Q1-belt). $PrgJ_1$ alpha helix 1 and the forks of $PrgJ_2$ and $PrgI_{27}$. Models are shown in ribbon cartoons with stick representations.

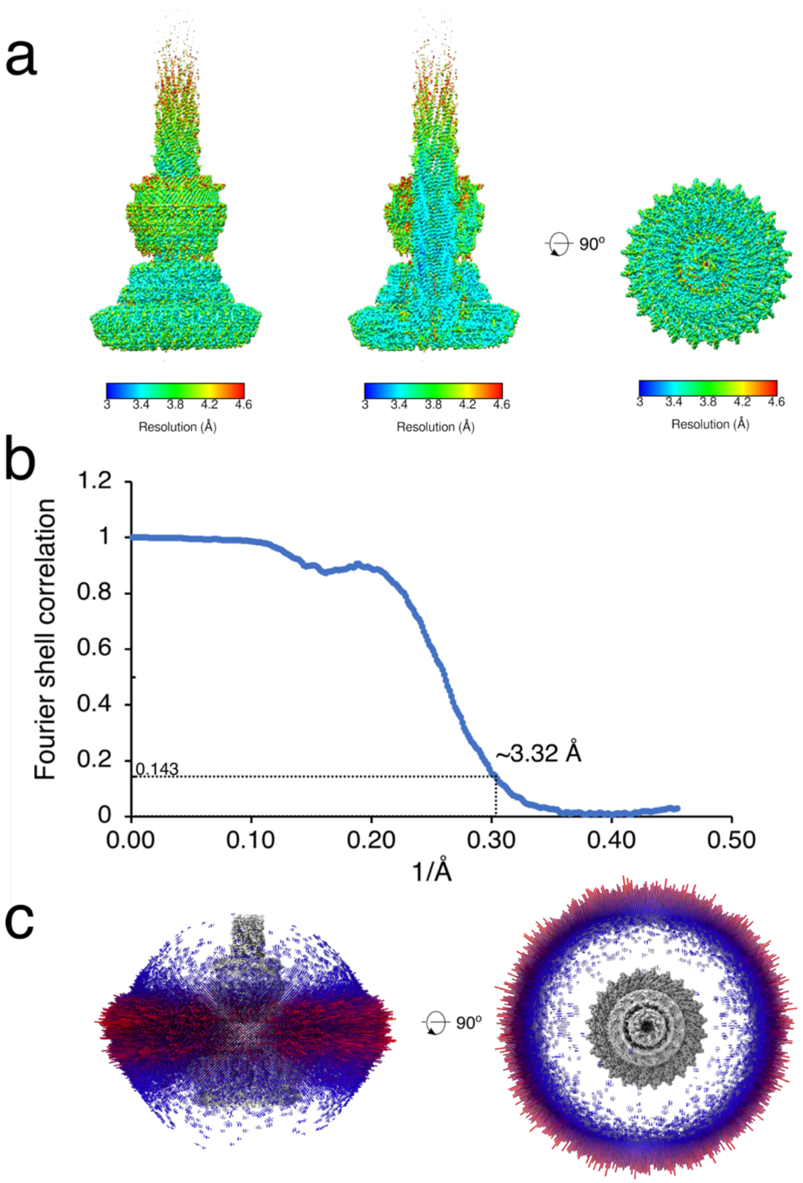

Supplementary Figure 5: Single particle reconstruction of the substrate-engaged needle complex from *S. enterica* sv. Typhimurium. a, Local resolution estimation of the masked C1 map (left), a vertical cross section (middle) and a top view (right) calculated by ResMap[1]. **b**, Fourier Shell Correlation (FSC) of the masked C1 map. Global resolution at the gold-standard FSC threshold of 0.143 is ~3.32Å. **c,** Side (left) and top (right) view of the angular distribution plot of all needle complex particles that contributed to the final C1 map. Height and color (from blue to red) of the cylinder bars is proportional to the number of particles.

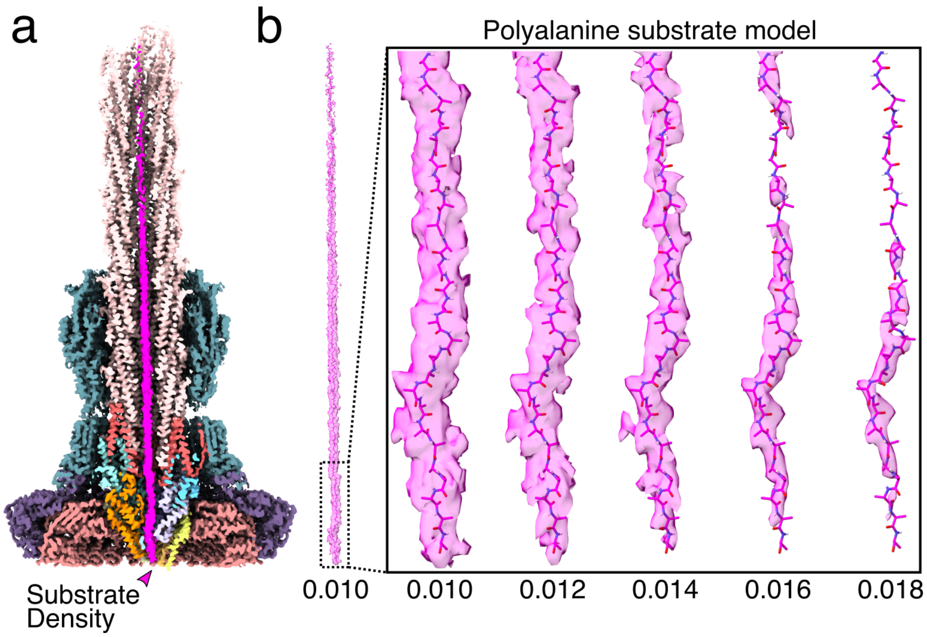

Supplementary Figure 6: The SptP3x-GFP substrate adopts a non-globular conformation inside the needle complex translocation channel. a, Cross section of the C1 map of the needle complex with the substrate density shown in magenta. **b**, varying thresholds of the substrate EM density, zoomed in on the length traversing the EA channel (box), with a modeled polyalanine sequence.

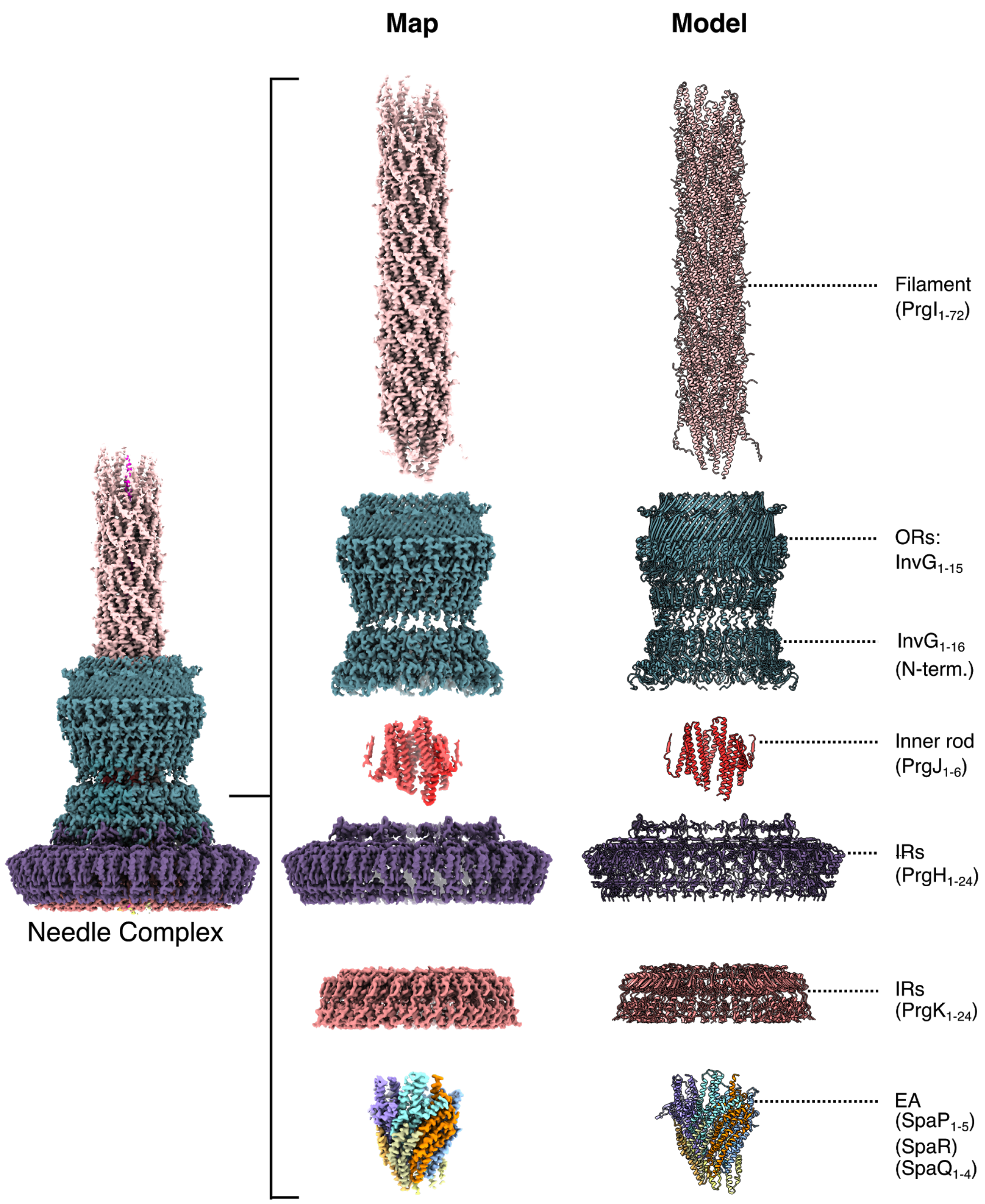

Supplementary Figure 7: Needle complex assembly. Extracted maps (left) and corresponding atomic models (right) of the export apparatus subcomplex containing SpaP, SpaQ and SpaR, the inner (IR; PrgK) and outer rings (OR; PrgH), the inner rod (PrgJ), the InvG ring with symmetry mismatch between the upper (C15) and lower (C16) OR component, and the filament (PrgI). Numbers correspond to the subunits present in each subcomplex.

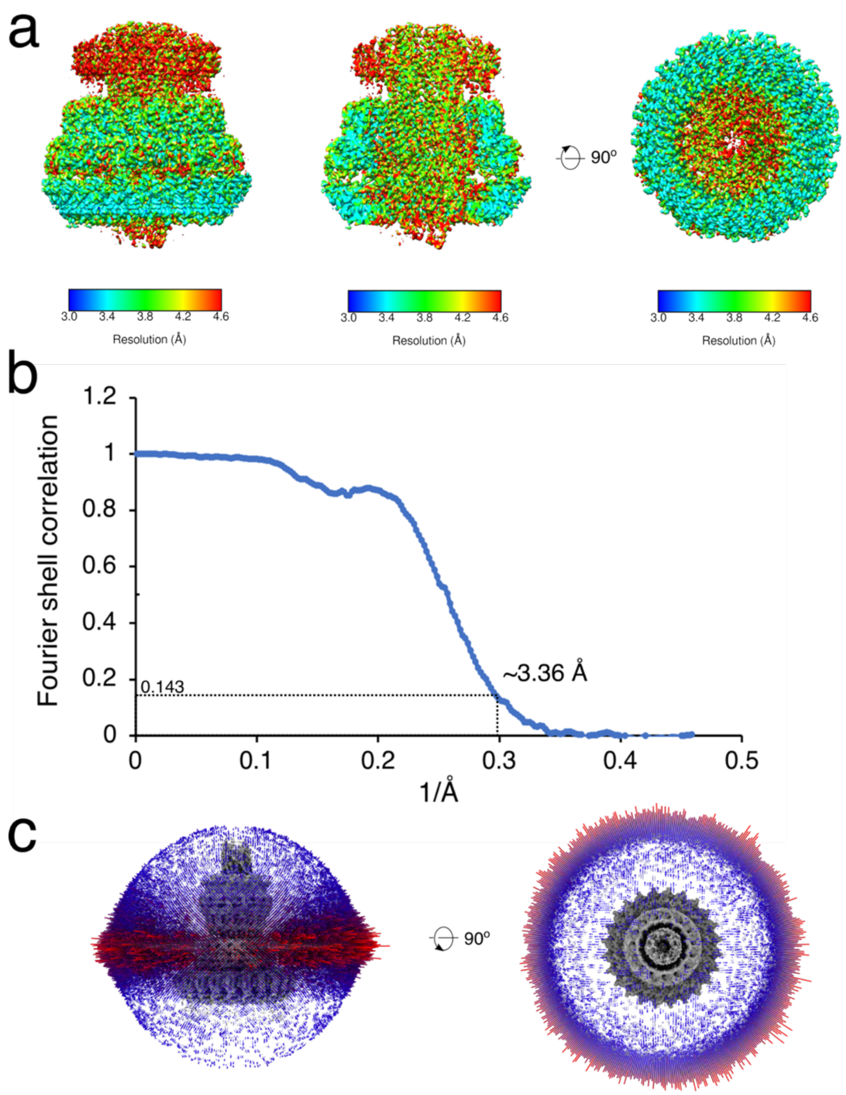

Supplementary Figure 8: Single particle reconstruction of the apo-state needle complex from *S. enterica* sv. Typhimurium. a, Local resolution estimation of the masked export apparatus and filament C1 map (left), a vertical cross section (middle) and a bottom view (right) calculated by ResMap[1]. **b,** Fourier Shell Correlation (FSC) of the masked C1 map. Global resolution at the gold-standard FSC threshold of 0.143 is ~3.36Å. **c**, Side (left) and top (right) view of the angular distribution plot of all needle complex particles that contributed to the final C1 map. Height and color (from blue to red) of the cylinder bars is proportional to the number of particles.

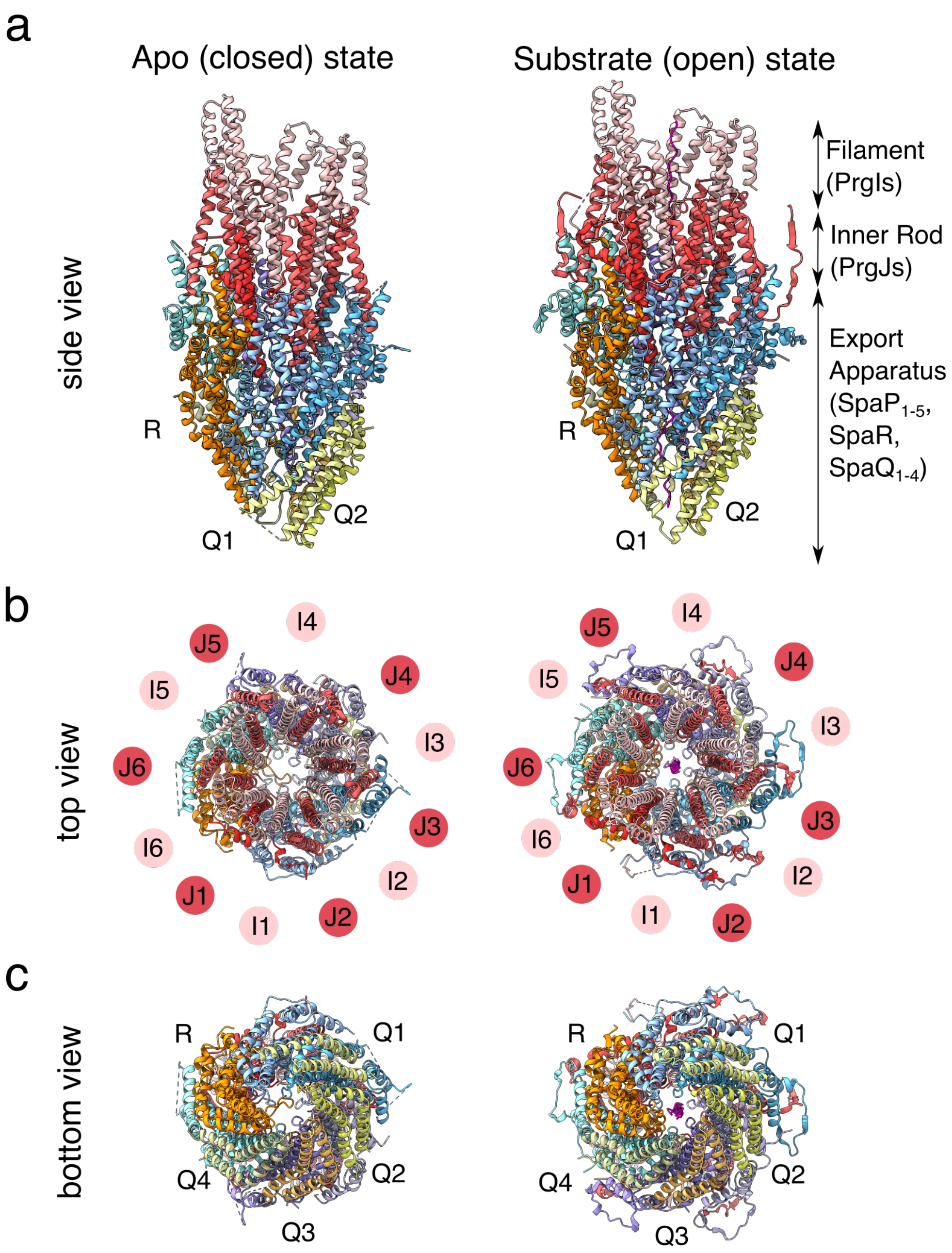

Supplementary Figure 9: Comparison of apo (closed) and substrate-engaged (open) EA complexes with assembled inner rod and filament base. a, Side views. **b**, Top views. **c**, Bottom views. Color Code: SpaQ (Q1-Q4, yellow colors), SpaR (R, orange), SpaP (P1-P5, blue colors), PrgJ (J1-J6, red colors), PrgI (I1-I6, pale pink), SptP3x-GFP substrate (magenta).

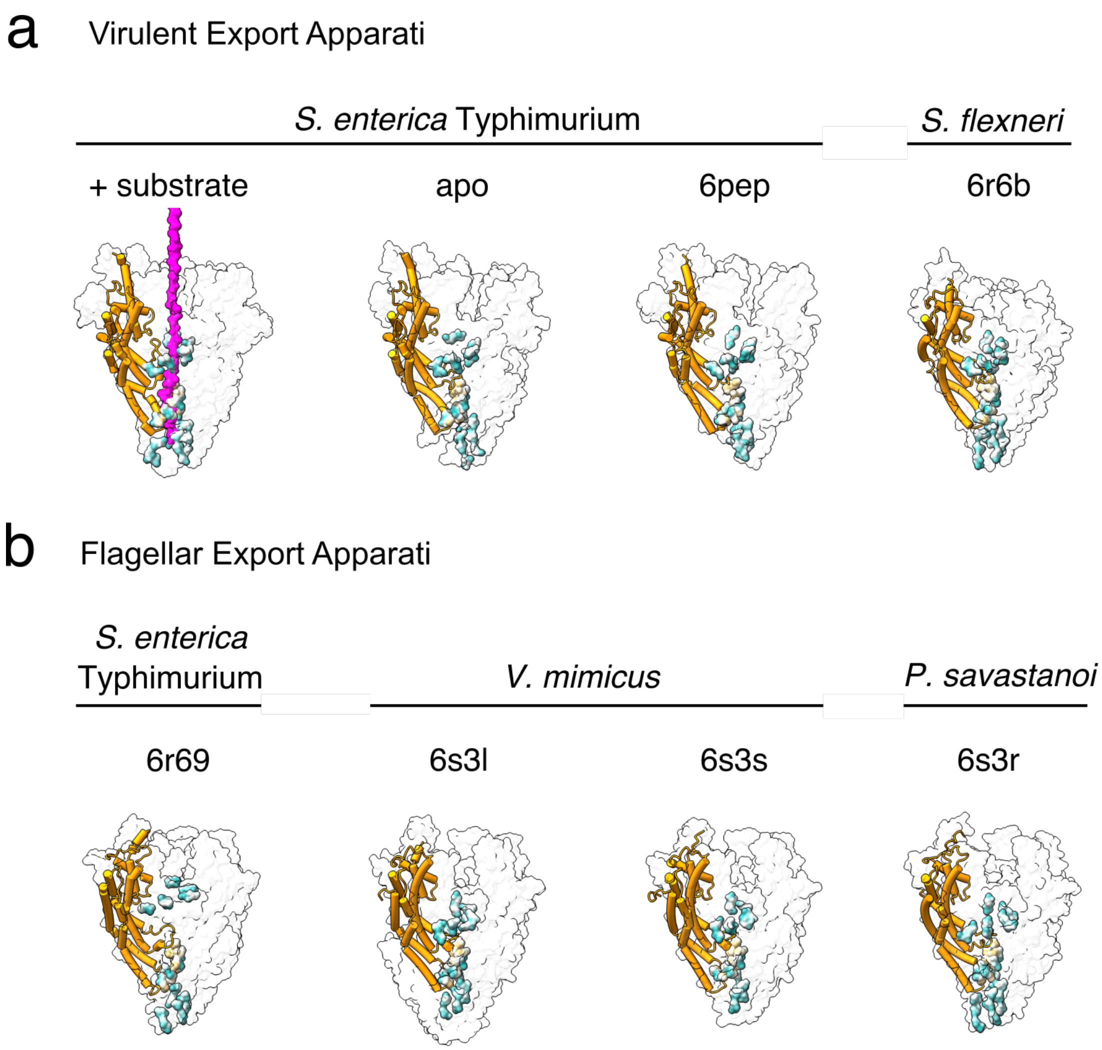

Supplementary Figure 10: Conserved Q1-belt/M-gate/Q2-belt architecture in EAs from virulent and flagellar T3SSs. a, Transparent surface representations of virulent EA proteins with SpaR homologues shown as cylinders colored in orange. Side chain surfaces of residues shaping the three EA interfaces, Q1-belt, M-gate and Q2-belt are highlighted where color represents hydrophobicity (green: hydrophilic; white: neutral; gold: hydrophobic). **b**, Representation style as in (a) but for published structures of flagellar EAs. SpaR homologue FliR colored in orange. Labels are PDB accession codes.

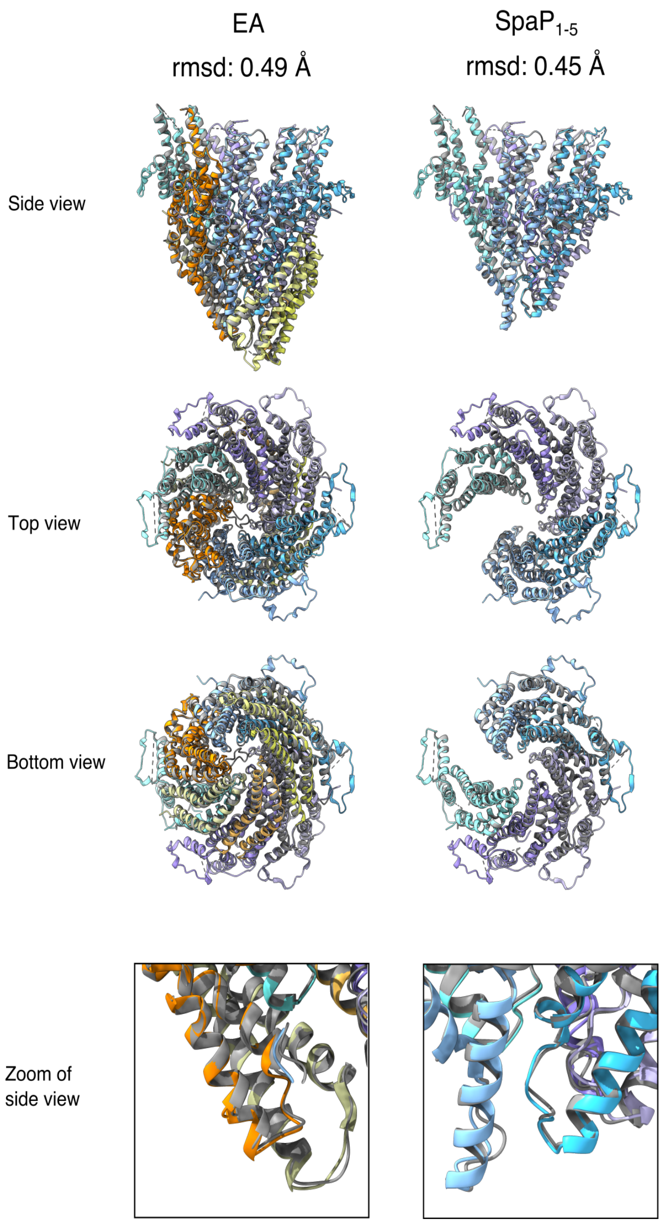

Supplementary Figure 11: Superposition of closed (apo-state) and open (substrate-engaged) EA structures. Proteins shown in ribbon diagrams with the apo-state EA colored in grey. Proteins of substrate-engaged EA are colored in orange (SpaR), blue colors ($SpaP_{1-5}$) and yellow colors ($SpaQ_{1-4}$). EA (SpaP:Q:R) complexes align with a root mean square deviation (rmsd) of 0.49Å (left). $SpaP_{1-5}$ align with a rmsd of 0.45Å (right).

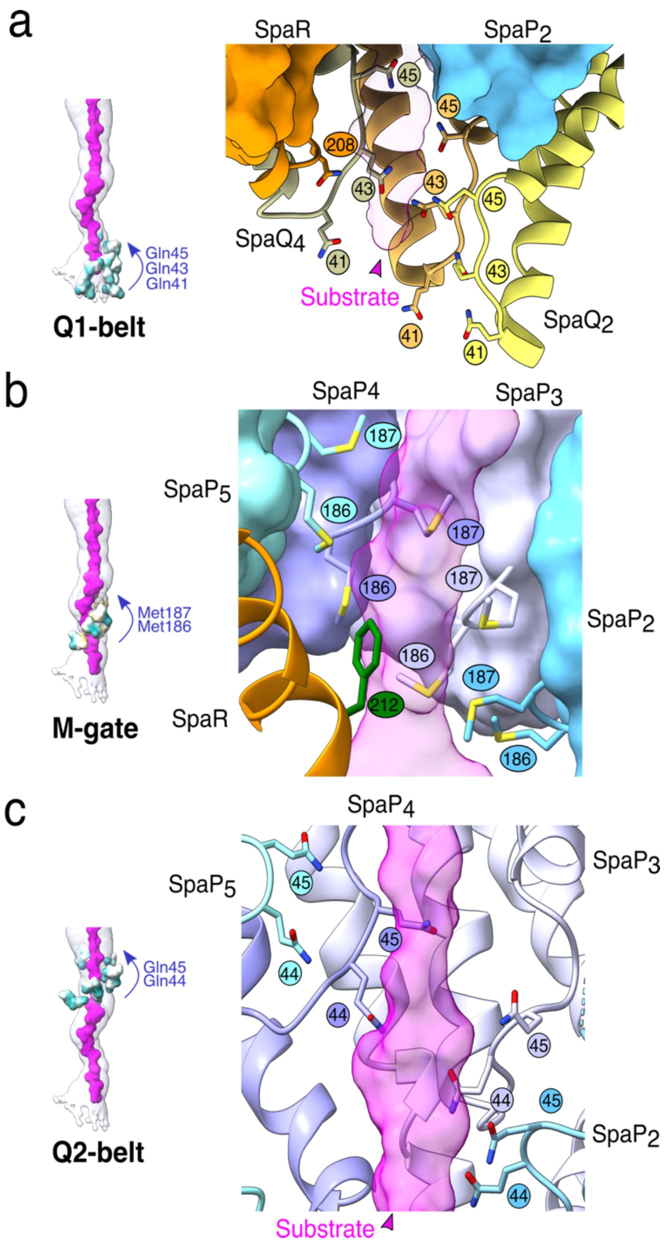

Supplementary Figure 12. Labelled residues of the injectisome EA Q1-belt, M-gate and Q2-belt. a, Gln41, Gln43 and Gln45 residues forming the SpaQ Q1-belt are labeled and shown as sticks. SpaQ$_1$ is omitted to aid visualization. **b,** SpaP residues Met186-187 forming the SpaP M-gate are labeled and shown as sticks. SpaR Phe212 is shown in green. SpaP$_1$ is omitted. **c,** Gln44-45 forming the SpaP Q2-belt are labelled and shown as sticks. SpaP$_1$ is omitted.

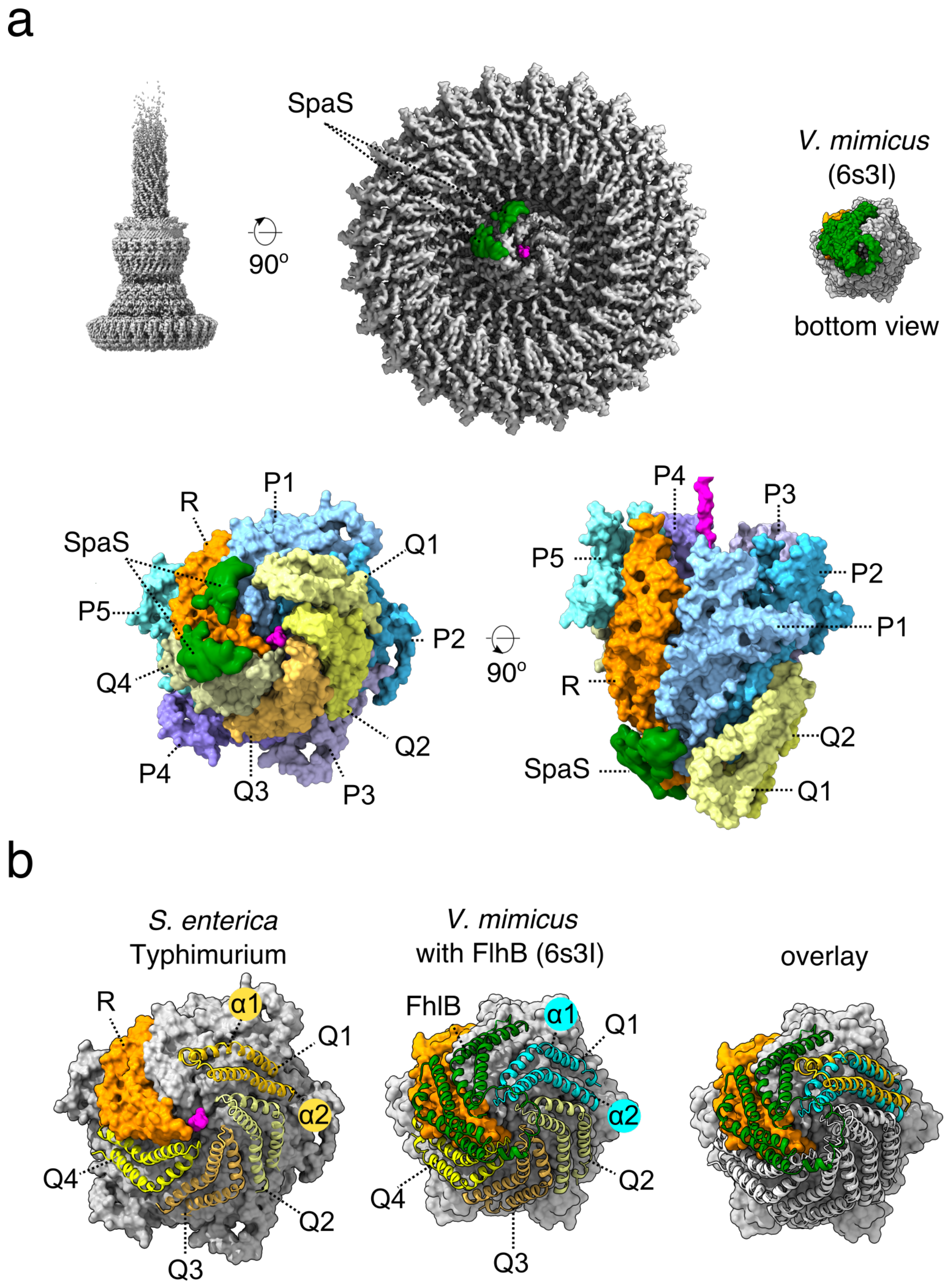

Supplementary Figure 13: Q1-belt portal of the EA contains SpaS and has a displaced $SpaQ_1$ when compared to the flagellar homologue from *Vibrio mimicus*. **a**, EM density (green) corresponding to SpaS N-terminal helices seen in the substrate-engaged needle complex map at high threshold (middle) and the recombinant EA complex (6s3l) from *V. mimicus* (right)[2]. Density was segmented from a smoothed map using Chimera Segger and placed on the map of the needle

complex[3,4]. Below, SpaS density placed on the SptP3x-GFP-containing EA model shown in surface representation. Color code: SpaP (blue, P1-5), SpaR (orange), SpaQ (yellow, Q1-4). **b**, Left: Surface representation of EAs with SpaP proteins colored in grey and SpaR in orange. SpaQ_{1-4} depicted as cartoon ribbons (yellow). Surface of the substrate model is displayed in magenta. α-helices of SpaQ_1 are labeled. Middle: Depiction of *V. mimicus* flagellar EA from Kuhlen *et al*., 2020 (PDB: 6s3l) with surfaces of FliPs (SpaP homologue) and FliR (SpaR homologue) shown in grey and orange, respectively[2]. FliQs (SpaQ homologue) depicted as yellow cartoon ribbons. α-helices of FliQ_1 are labeled. Right: Superposition of SpaQs from substrate-engaged EA complex onto the *V. mimicus* EA with corresponding FliQ_1 highlighted in cyan. SpaQ_{1-4} and FliQ_{1-4} superimpose with a root mean square deviation (rmsd) of 1.27Å.

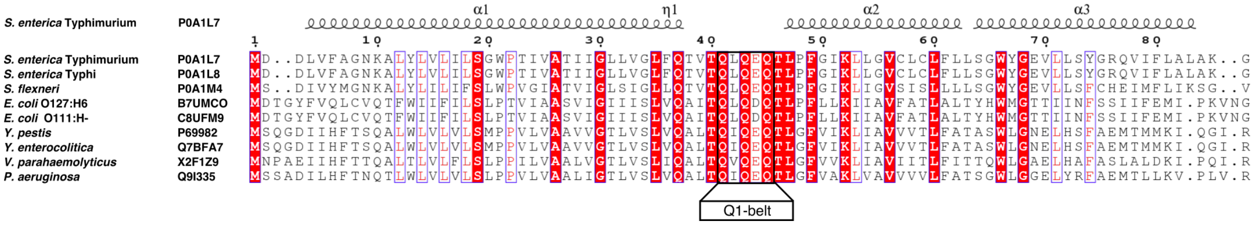

Supplementary Figure 14: Multiple sequence alignment of SpaQ homologues. *S. enterica* sv. Typhimurium (P0A1L7), *S. enterica* sv. Typhi (P0A1L8), *S. flexneri* (P0A1M4), EPEC (B7UMC0), EHEC (C8UFM9), *Y. pestis* (P69982), *Y. enterocolitica* (Q7BFA7), *V. parahaemolyticus* (X2F1Z9), *P. aeruginosa* (Q9I335). Uniprot accession numbers indicated. Residues involved in shaping the Q1-belt in SpaQ in *S. enterica* sv. Typhimurium (Gln41, Gln43 and Gln45) and other homologues are boxed out. Alignment performed using T-Coffee and displayed with EsPript 3.0 server[5,6].

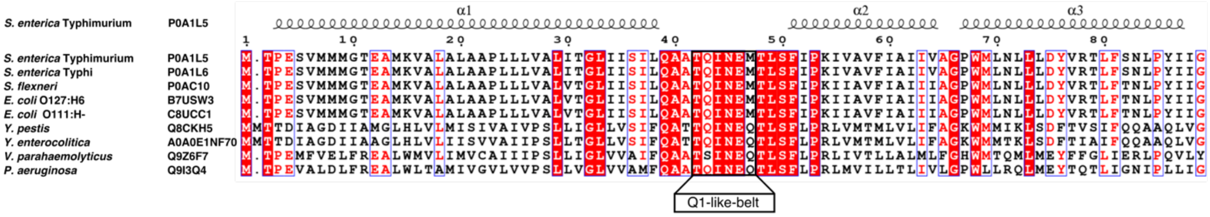

Supplementary Figure 15: Multiple sequence alignment of FliQ homologues. *S. enterica* sv. Typhimurium (P0A1L5), *S. enterica* sv. Typhi (P0A1L6), *S. flexneri* (P0AC10), EPEC (B7USW3), EHEC (C8UCC1), *Y. pestis* (Q8CKH5), *Y. enterocolitica* (A0A0E1NF70), *V. parahaemolyticus* (Q9Z6F7), *P. aeruginosa* (Q9I3Q4). Uniprot accession numbers indicated. Residues involved in shaping a hydrophilic Q1-like-belt in FliQ in *S. enterica* sv. Typhimurium and other homologues are boxed out. Alignment performed using T-Coffee and displayed with EsPript 3.0 server[5,6].

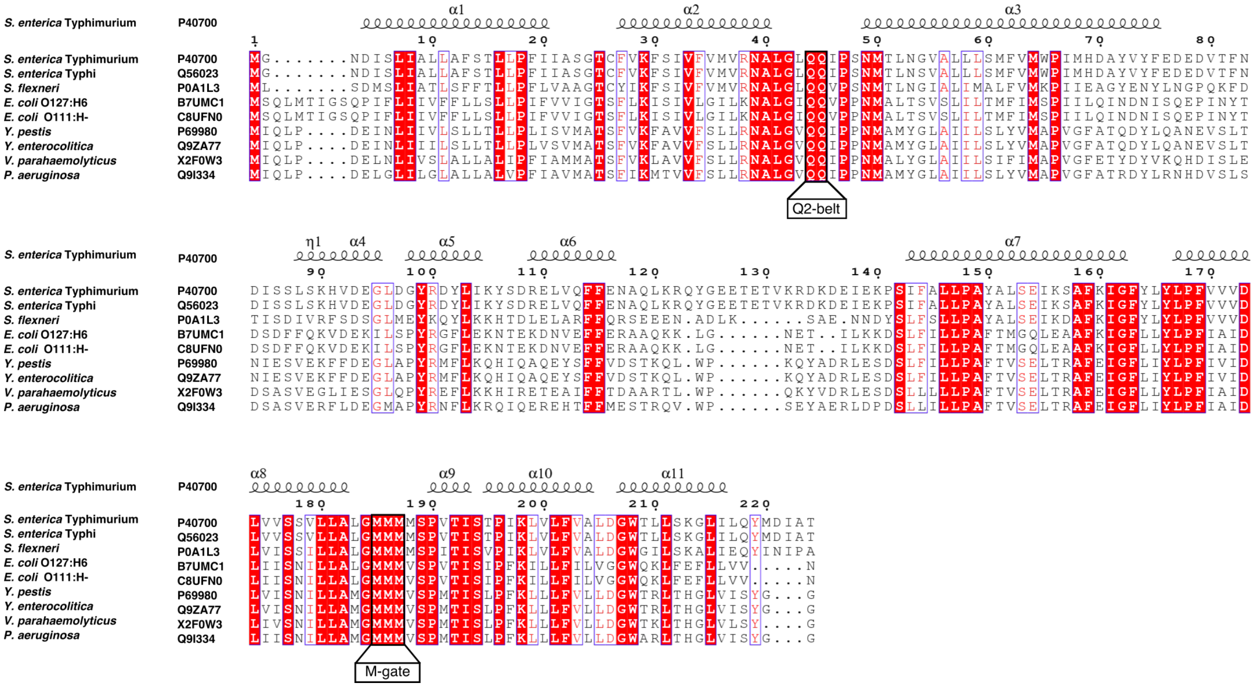

Supplementary Figure 16: Multiple sequence alignment of SpaP homologues. *S. enterica* sv. Typhimurium (P40700), *S. enterica* sv. Typhi (Q56023), *S. flexneri* (P0A1L3), EPEC (B7UMC1), EHEC (C8UFN0), *Y. pestis* (P69980), *Y. enterocolitica* (Q9ZA77), *V. parahaemolyticus* (X2F0W3), *P. aeruginosa* (Q9I334). Uniprot accession numbers are indicated. Residues involved in shaping the Q2-belt and M-gate in SpaP in *S. enterica* sv. Typhimurium (Gln44, Gln45 and Met185-187) and other homologues are boxed out. Alignment performed using T-Coffee, and displayed with EsPript 3.0 server[5,6].

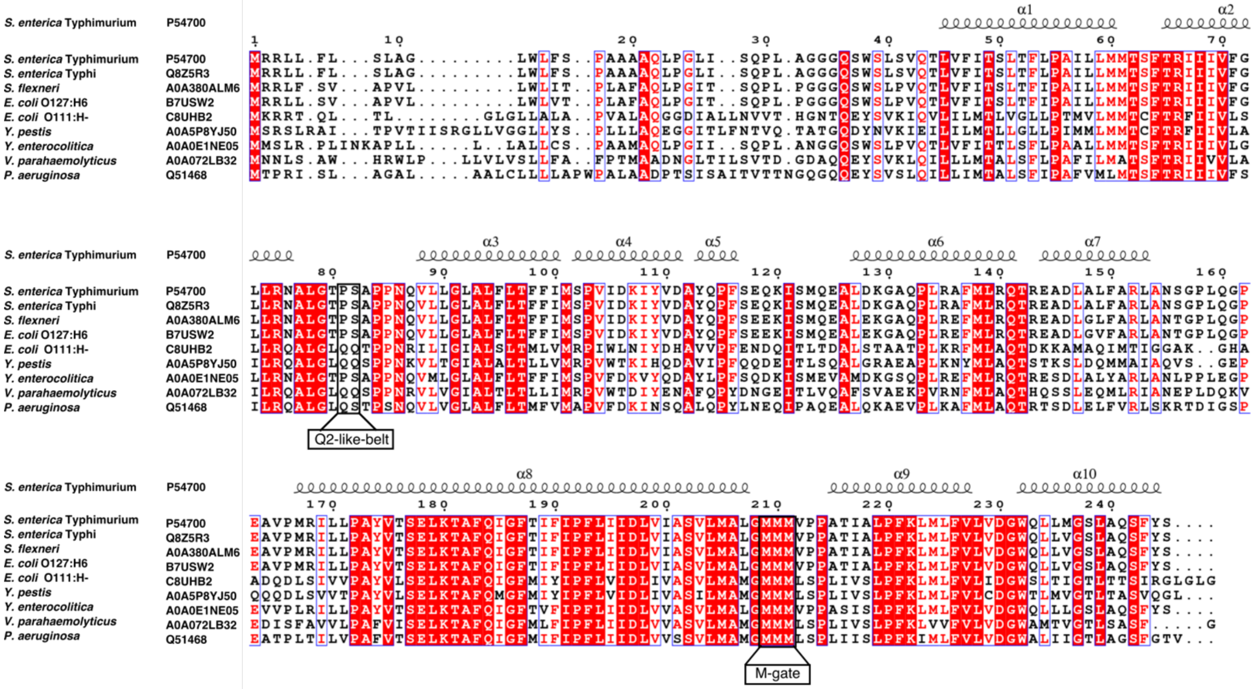

Supplementary Figure 17: Multiple sequence alignment of FliP homologues. *S. enterica* sv. Typhimurium (P40700), *S. enterica* sv. Typhi (Q8Z5R3), *S. flexneri* (A0A380ALM6), EPEC (B7USW2), EHEC (C8UHB2), *Y. pestis* (A0A5P8YJ50), *Y. enterocolitica* (A0A0E1NE05), *V. parahaemolyticus* (A0A072LB32), *P. aeruginosa* (Q51468). Uniprot accession numbers are indicated. Residues involved in shaping a hydrophilic Q2-like-belt and M-gate in FliP in *S. enterica* sv. Typhimurium (Pro81, Ser82 and Met209-211) and other homologues are boxed out. Alignment performed using T-Coffee, and displayed with EsPript 3.0 server[5,6].

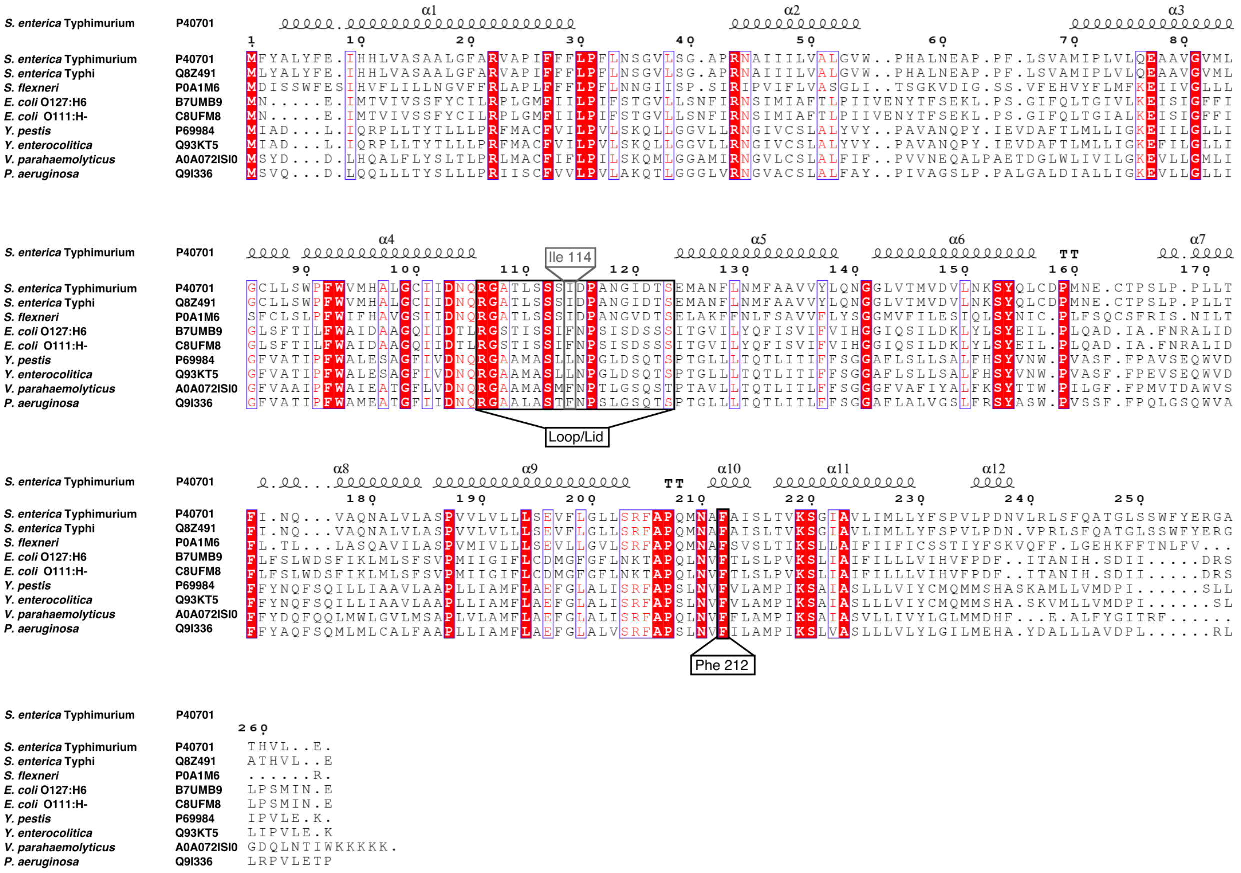

Supplementary Figure 18: Multiple sequence alignment of SpaR homologues. *S. enterica* sv. Typhimurium (P40701), *S. enterica* sv. Typhi (Q8Z491), *S. flexneri* (P0A1M6), EPEC (B7UMB9), EHEC (C8UFM8), *Y. pestis* (P69984), *Y. enterocolitica* (Q93KT5), *V. parahaemolyticus* (A0A072ISI0), *P. aeruginosa* (Q9I336). Uniprot accession numbers are indicated. Residues involved in shaping the loop/lid, Ile114, and Phe212 of SpaR in *S. enterica* sv. Typhimurium and other homologues, are boxed out. Alignment performed using T-Coffee and displayed with EsPript 3.0 server[5,6].

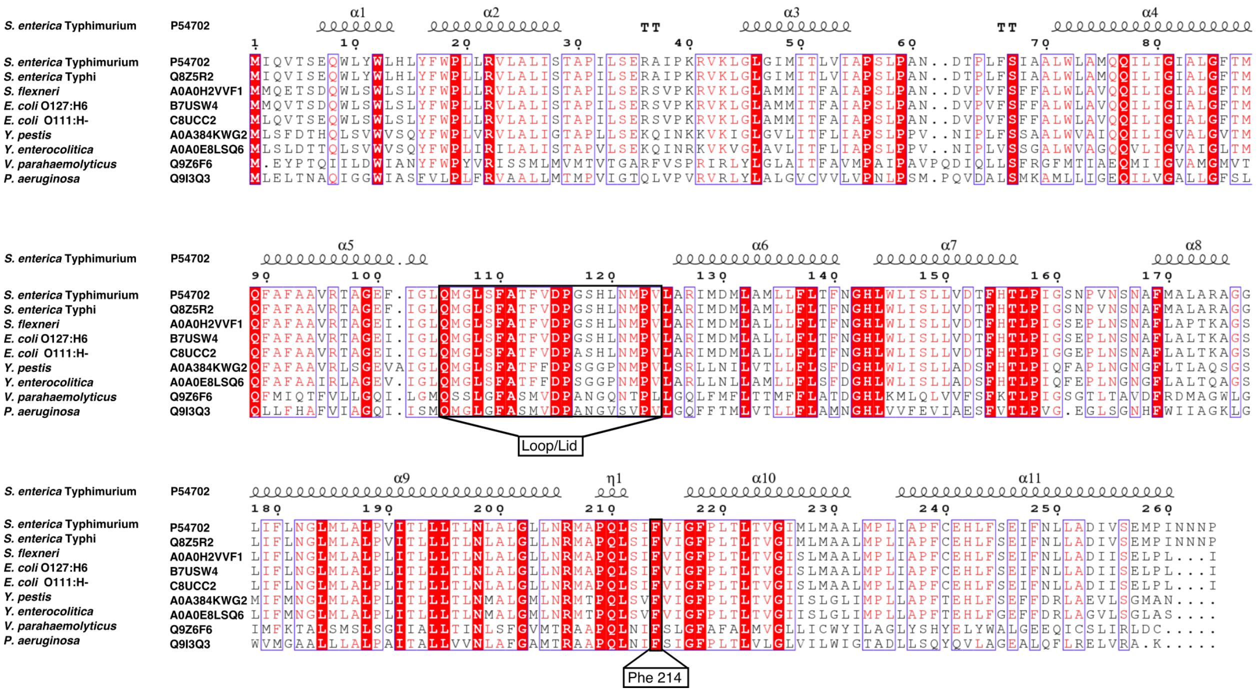

Supplementary Figure 19: Multiple sequence alignment of FliR homologues. *S. enterica* sv. Typhimurium (P54702), *S. enterica* sv. Typhi (Q8Z5R2), *S. flexneri* (A0A0H2VVF1), EPEC (B7USW4), EHEC (C8UCC2), *Y. pestis* (A0A384KWG2), *Y. enterocolitica* (A0A0E8LSQ6), *V. parahaemolyticus* (Q9Z6F6), *P. aeruginosa* (Q9I3Q3). Uniprot accession numbers are indicated. Residues involved in shaping the FliR loop/lid and Phe214 are boxed out and correspond to the homologous loop/lid and Phe212 of SpaR in *S. enterica* sv. Typhimurium. In FliR, Phe110 and Phe113 of the loop form contacts with the M-gate below. Alignment performed using T-Coffee and displayed with EsPript 3.0 server[5,6].

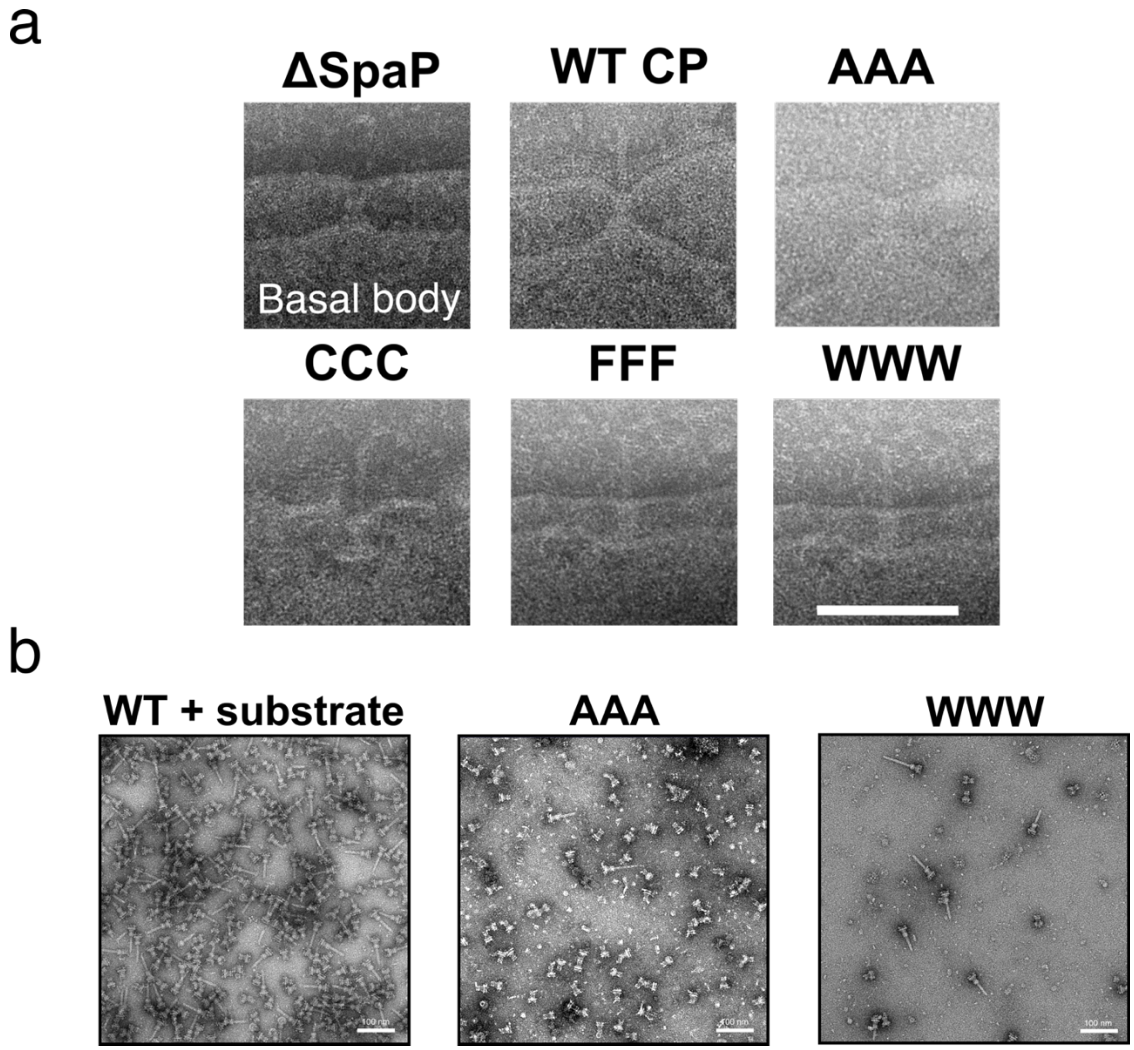

Supplementary Figure 20: Visualization of needle complexes in *Salmonella* SpaP M-gate mutants. a, Representative micrographs of basal bodies (ΔSpaP) and needle complexes (AAA, CCC, FFF, WWW) in osmotically-shocked cells from SpaP knockout strains complemented with the corresponding SpaP M-gate mutations. Scale bar shown in the WWW micrograph is 100 nm and corresponds to all micrographs in (a). Images are representative of one of three independent experiments. **b**, Micrographs of needle complex purifications from *Salmonella* expressing the substrate SptP3x-GFP (left) or $SpaP^{KO}$ knockout strain complemented with $SpaP^{AAA}$ (middle) or $SpaP^{WWW}$ (right). Samples were imaged via negative stain TEM using 2% w/v PTA. Needle-less basal bodies correspond to incomplete assembly states of needle complexes.

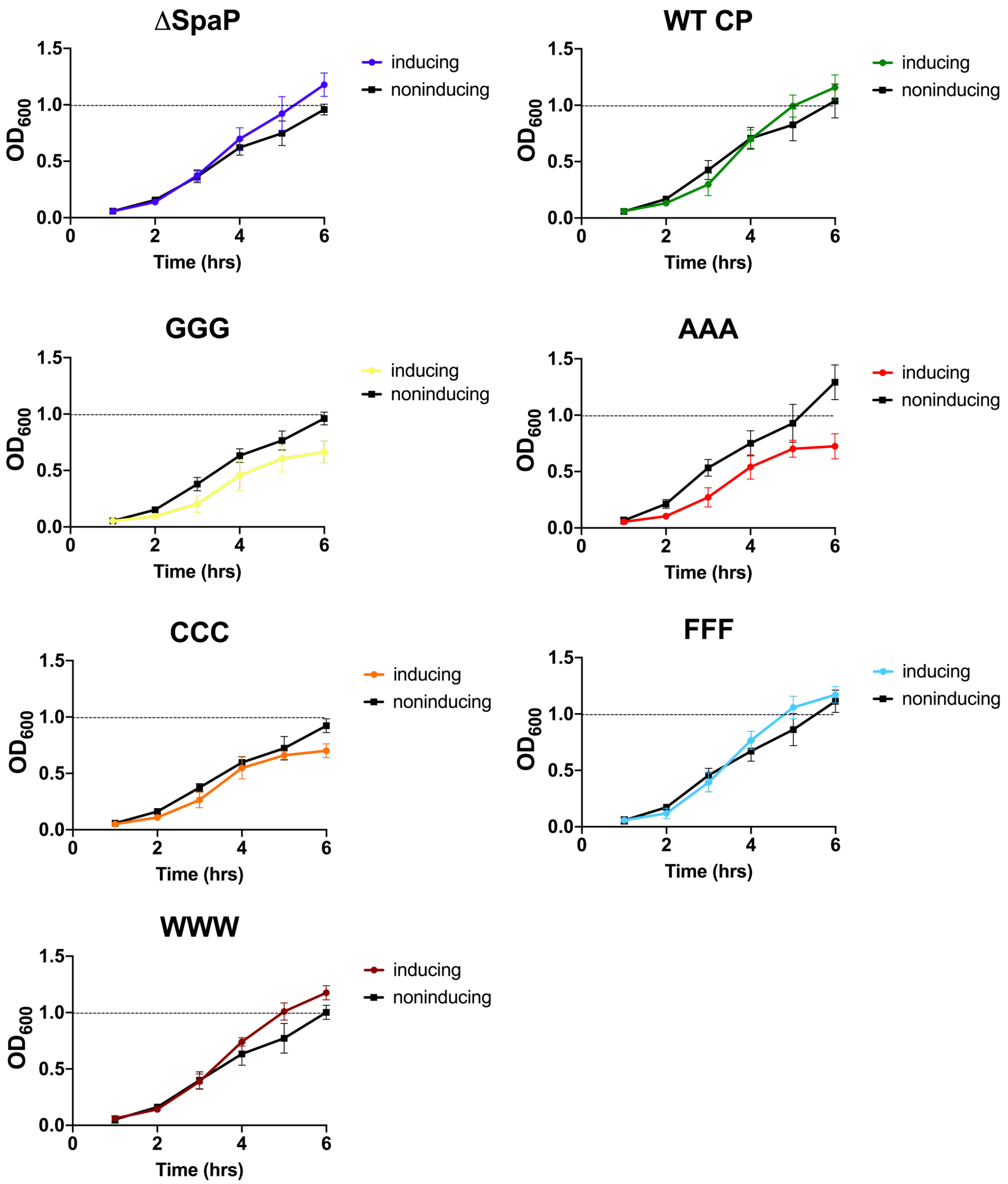

Supplementary Figure 21: Growth curves of *Salmonella* SpaP knockout strains complemented with SpaP harboring M-gate mutations. Each chart plots OD_{600} of a 6 h time course for each strain. All strains are SpaP knockout and WT CP is complemented with WT SpaP. Black and colored lines represent growth under non-inducing and inducing conditions, respectively. Plotted values are the average from 9 independent measurements from 3 independent experiments. Error bars represent standard deviation (SD).

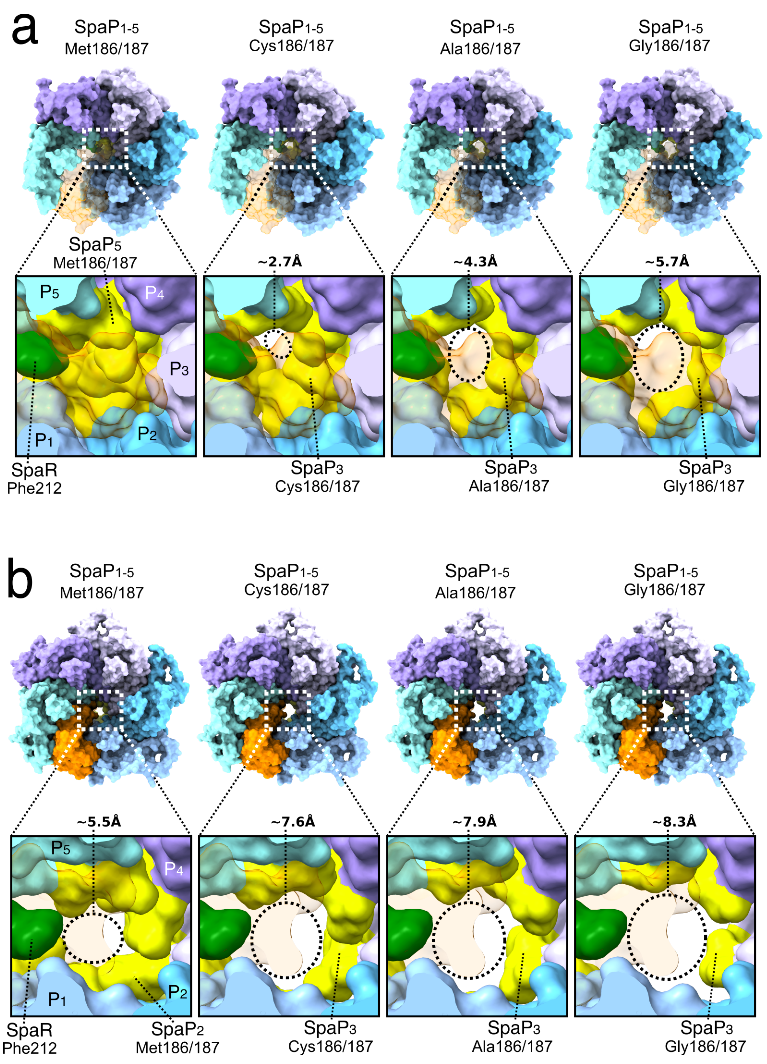

Supplementary Figure 22: Mutations of methionines 186 and 187 to cysteines, alanines or glycines increase the pore size of the M-gate. a, Top views onto the apo-state EA complex with SpaP$_{1-5}$ Met186 and Met187 mutated to either cysteine, alanine or glycine. Approximate diameter of the M-gates were calculated from modeled surfaces. Surface of SpaR (with the exception of SpaR Phe212 shown in green) is transparent to aid visualization of the M-gate. Residues 186 and 187 are colored in yellow. **b**, Top views onto the substrate-state EA complex with SpaP$_{1-5}$ mutated to either cysteine, alanine or glycine. Approximate diameter of the M-gates were calculated from modeled surfaces. The substrate has been removed to aid visualization.

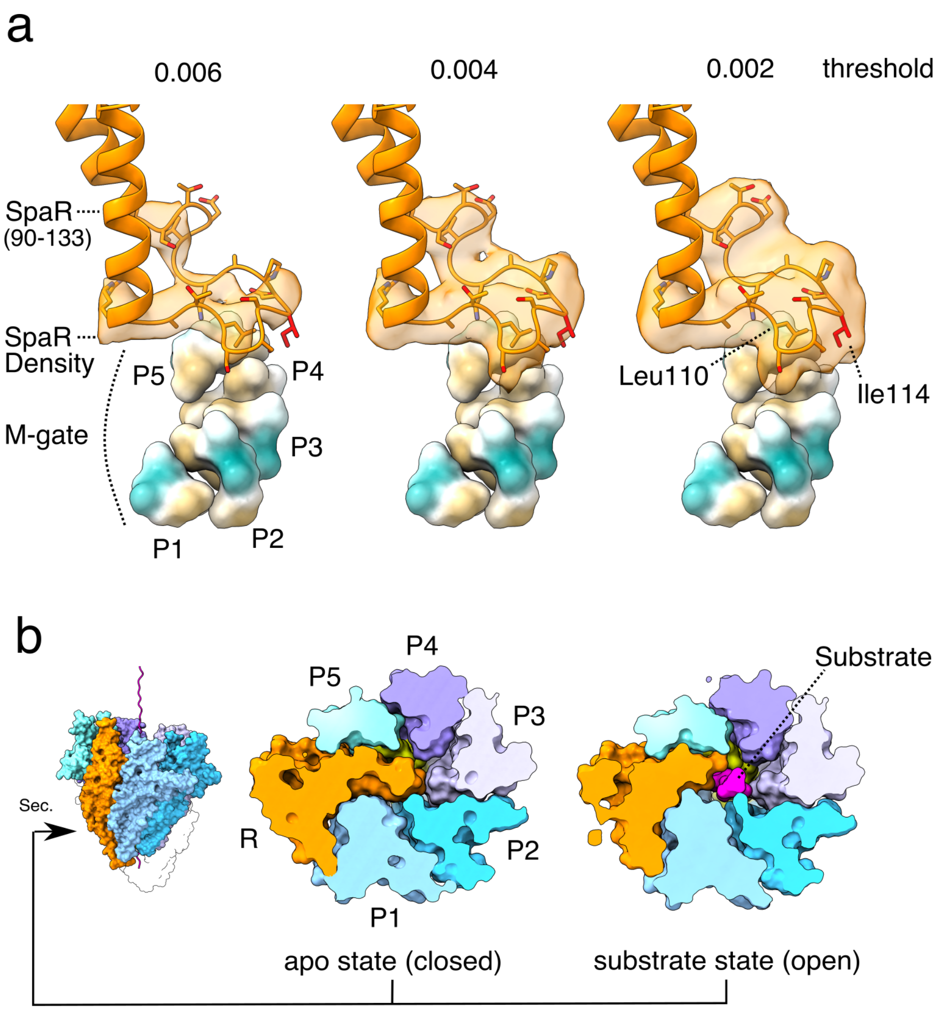

Supplementary Figure 23: A unique loop of residues 106-123 of SpaR seals the EA translocation channel. a, The closed SpaR loop/lid is shown with a filtered density (B-factor of 100) and modeled residues. The SpaP M-gate methionine residues are shown below as surface representations and colored by hydrophobicity. **b,** Left: overview of the EA with the horizontal position marked corresponding to cross sections of the SpaR loop through the apo-state EA complex (middle) and substrate-engaged EA complex (right). Proteins are depicted as surface representations. SpaP$_{1-4}$ is depicted in blue, SpaR in orange, and SpaQ$_{1-4}$ is transparent. The substrate is depicted in magenta and the M-gate methionines are colored yellow.

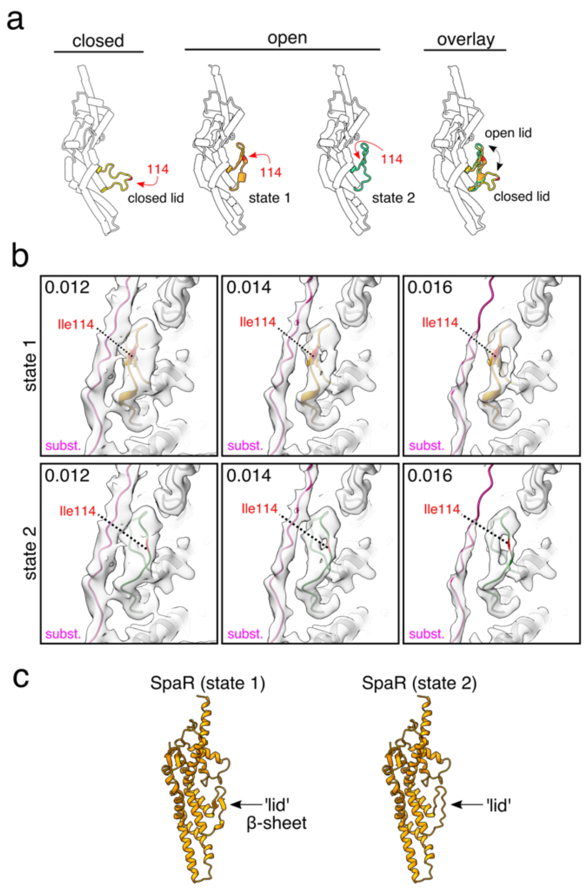

Supplementary Figure 24: SpaR loop 106-123 forms two distinct conformations. a, Transparent cylinder representation of SpaR with its lid in closed (left; yellow) or two distinct open conformations (middle; state 1 is colored orange, state 2 is green). Overlay between all three conformations (right) illustrates the movement of the loop from a horizontal (closed state) to vertical positions (open states). **b**, Panels show EM densities with increasing map threshold levels (numbers). Model is depicted in ribbon diagram representation with the substrate (subst.) shown in magenta and SpaR loop conformational states one and two in orange or green, respectively. Position of Ile114 is highlighted in red. **c**, Ribbon diagrams of SpaR with loop residues 106-123 in the two different conformations where in state 1, the SpaR loop is stabilized by a short antiparallel β-sheet.

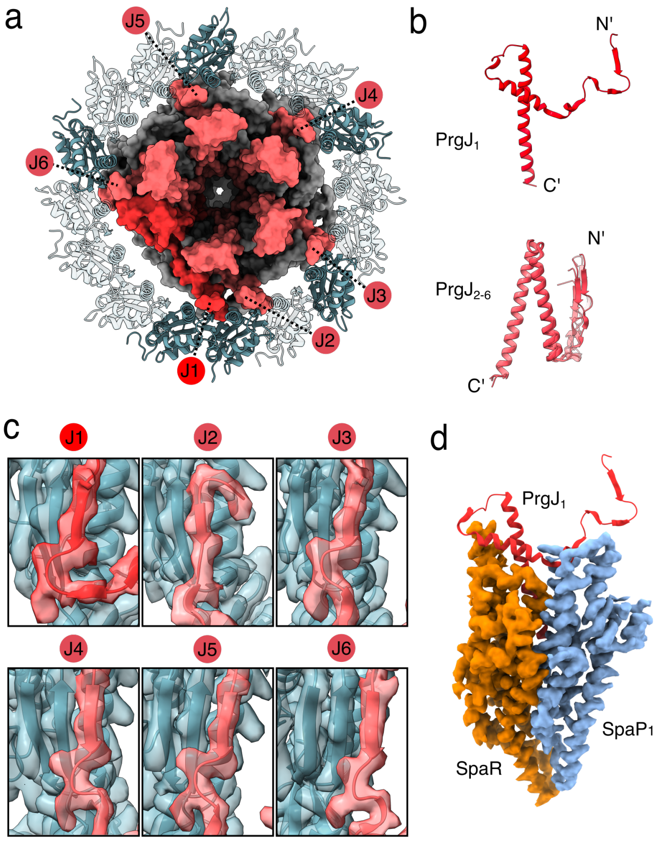

Supplementary Figure 25: PrgJ proteins contact EA and InvG proteins to stabilize the inner rod in the confinement of the basal body. a, Top view of the EA and inner rod together in surface representation and with the lower part of the InvG 16-mer ring in ribbon diagram representation (also see Supplementary Fig. 7). InvG monomers forming contacts with $PrgJ_{1-6}$ N-termini are colored dark green. **b,** Comparison of the unique fold of $PrgJ_1$ (top) and superimposed $PrgJ_{2-6}$ (bottom) shown as ribbon diagrams. **c,** Close up views of $PrgJ_{1-6}$ N-termini forming β-sheet complementation with subunits of the InvG 16-mer ring, as shown in (**a**), depicted as ribbon diagrams with the corresponding EM density. **d,** The unique fold of $PrgJ_1$ shown as a red ribbon diagram, interfacing SpaR shown as EM density in orange and traversing $SpaP_1$ also shown as EM density in blue.

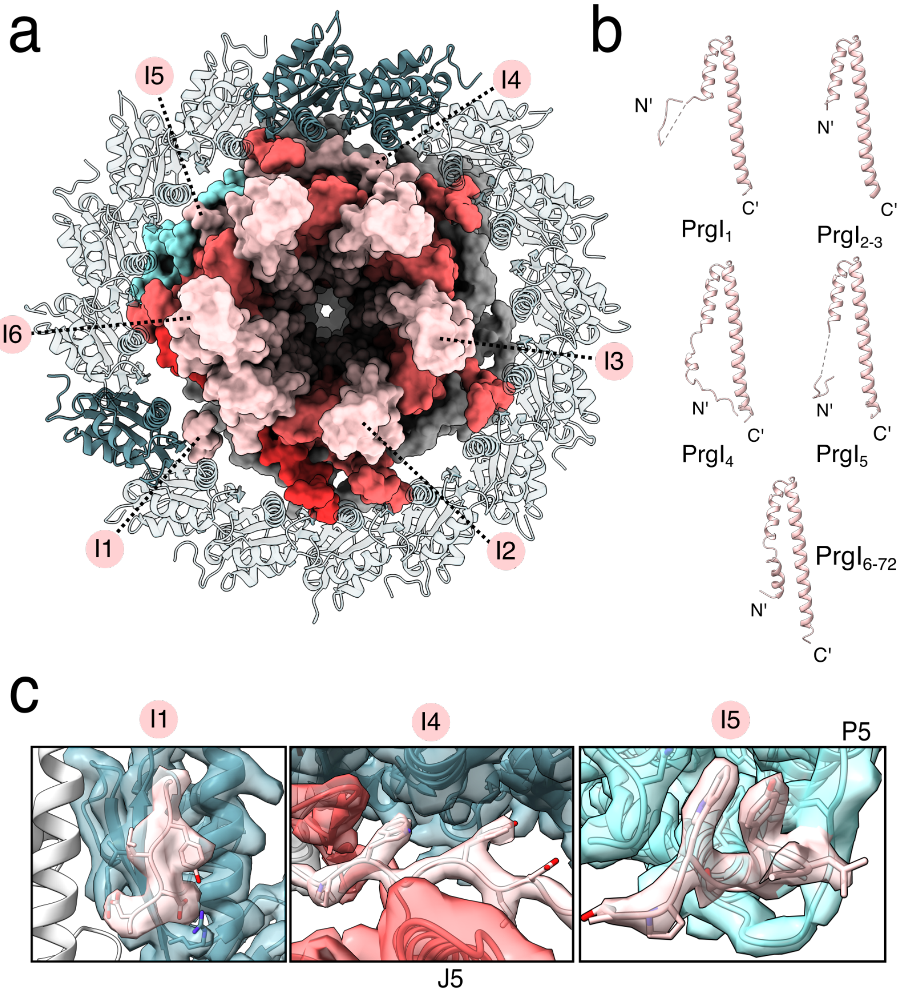

Supplementary Figure 26: PrgI proteins polymerize on top of the inner rod and contact EA and InvG proteins to stabilize the filament in the confinement of the basal body. a, Top view onto the EA with molecular surfaces of SpaP$_5$ in blue and SpaP$_{1\text{-}4}$ in grey, PrgJ in red and PrgI in salmon. InvG N-terminal domains are depicted as ribbon diagrams. InvG monomers forming contacts with PrgI$_{1,4,5}$ are colored in dark green **b**, Ribbon diagrams of PrgI proteins illustrating structural differences between PrgI$_1$, PrgI$_{2\text{-}3}$, PrgI$_4$, PrgI$_5$ and PrgI$_{6\text{-}72}$. **c**, Interfaces of PrgI$_{1,4\text{-}5}$ N-termini with InvG (PrgI$_1$, PrgI$_4$), PrgJ$_5$ (PrgI$_4$) and SpaP$_5$ (PrgI$_5$). Surfaces represent EM density.

Supplementary Tables

**Injectisome component**	**Protein nomenclature in *Salmonella* SPI-1**	**Unified nomenclature**
Basal body rings	InvG	SctC
	PrgH	SctD
	PrgK	SctJ
Export apparatus	SpaP	SctR
	SpaQ	SctS
	SpaR	SctT
	SpaS	SctU
	InvA	SctV
Inner rod	PrgJ	SctI
Filament	PrgI	SctF
Translocases + tip	SipB	SctE
	SipC	SctB
	SipD	SctA
Sorting platform	OrgA	SctK
	SpaO	SctQ
	OrgB	SctL
	InvC	SctN
	InvI	SctO

Supplementary Table 1. Protein components of the *Salmonella* SPI-1 injectisome and the corresponding unified T3SS nomenclature[7].

Structure	Conformational state 1	Conformational state 2
interface area	679Å	694Å2
ΔG:	-4.4 kcal/mol	-4.1 kcal/mol
ΔG p-value	0.86	0.85
Complexation Significance score	0	0

Supplementary Table 2. PDBePISA analysis of SpaR loop/lid conformational states 1 and 2[8].

Salmonella enterica Strain	Description	Parent strain	Reference	Resistance	Transformed plasmids
SB905	wild type strain, flagellar knockout	SJW2941	Sukhan et al., 2001[9]	CmR	pSB1418-HilA
SB905 + substrate	wild type strain, flagellar knockout, expresses substrate	SB905	Radics et al., 2014[10]	CmR, AmpR	pSB3291-HilA, pACYCDuet-3-SptP3x-GFP-SicP
ΔSpaP	Deletion of *SpaP* gene	SB905	This study	CmR	pSB1418-HilA
SpaP$^{WT\ CP}$	Complemented with WT SpaP	ΔSpaP	This study	CmR, AmpR	pSB1418-HilA, pWSK29-SpaP
SpaP^{AAA}<br>SpaP^{GGG}<br>SpaP^{CCC}<br>SpaP^{FFF}<br>SpaP^{WWW}	Complemented with substitutions of SpaP residues Met185-7 (M-gate)				
SpaP^{AA}<br>SpaP^{GG}	Complemented with substitutions of SpaP residues Gln44-45 (Q2-belt)				
ΔSpaQ	Deletion of *spaQ* gene	SB905	This study	CmR	pSB1418-HilA
SpaQ$^{G\text{-}G\text{-}G}$<br>SpaQ$^{A\text{-}A\text{-}A}$<br>SpaQ$^{S\text{-}S\text{-}S}$	Genomic substitutions of SpaQ residues Gln41,43,45 (Q1-belt)				
ΔSpaR	Deletion of *spaR* gene				
SpaR^{I114A}	Genomic substitutions of SpaR residue Ile114 (loop/lid)				

Supplementary Table 3. Strains used in the study.

Plasmids	Description	Parent plasmid	Reference	Resistance
pSB1418-HilA	Encodes *hilA* gene - overexpression of the SPI-1	pBAD18	Marlovits et al., 2004[11]	CmR
pSB3291-HilA		pBR322 / pBAD18	Kubori et al., 1998[12]	AmpR
pACYCDuet-3-SptP3x-GFP-SicP	Encodes SptP3x-GFP substrate and SicP chaperone	pACYC	Radics et al., 2014[10]	CmR
pWSK29-SpaP	Complementation plasmid for SpaP, uses *prgH* gene promoter for expression	pSC101	This study	AmpR

Supplementary Table 4. Plasmids used in the study.

Supplementary References

1. Kucukelbir, A., Sigworth, F. J. & Tagare, H. D. Quantifying the local resolution of cryo-EM density maps. *Nat. Methods* **11**, 63–65 (2014).
2. Kuhlen, L. *et al.* The substrate specificity switch FlhB assembles onto the export gate to regulate type three secretion. *Nat. Commun.* **11**, 1–10 (2020).
3. Pintilie, G., Zhang, J., Chiu, W. & Gossard, D. Identifying components in 3D density maps of protein nanomachines by multi-scale segmentation. *IEEENIH Life Sci. Syst. Appl. Workshop IEEENIH Life Sci. Syst. Appl. Workshop* **2009**, 44–47 (2009).
4. Pintilie, G. & Chiu, W. Comparison of Segger and other methods for segmentation and rigid-body docking of molecular components in cryo-EM density maps. *Biopolymers* **97**, 742–760 (2012).
5. Madeira, F. *et al.* The EMBL-EBI search and sequence analysis tools APIs in 2019. *Nucleic Acids Res.* **47**, W636–W641 (2019).
6. Robert, X. & Gouet, P. Deciphering key features in protein structures with the new ENDscript server. *Nucleic Acids Res.* **42**, W320–W324 (2014).
7. Hueck, C. J. Type III protein secretion systems in bacterial pathogens of animals and plants. *Microbiol. Mol. Biol. Rev. MMBR* **62**, 379–433 (1998).
8. Krissinel, E. & Henrick, K. Inference of macromolecular assemblies from crystalline state. *J. Mol. Biol.* **372**, 774–797 (2007).
9. Sukhan, A., Kubori, T., Wilson, J. & Galán, J. E. Genetic Analysis of Assembly of the *Salmonella enterica* Serovar Typhimurium Type III Secretion-Associated Needle Complex. *J. Bacteriol.* **183**, 1159–1167 (2001).

10. Radics, J., Königsmaier, L. & Marlovits, T. C. Structure of a pathogenic type 3 secretion system in action. *Nat. Struct. Mol. Biol.* **21**, 82–87 (2014).

11. Marlovits, T. C. *et al.* Structural insights into the assembly of the type III secretion needle complex. *Science* **306**, 1040–2 (2004).

12. Kubori, T. *et al.* Supramolecular structure of the *Salmonella typhimurium* type III protein secretion system. *Science* **280**, 602–605 (1998).
