# Supplementary material for: Substrate-engaged type III secretion system structures reveal gating mechanism for unfolded protein translocation
Source: Nat Commun. 2021 Mar 9;12:1546. doi: 10.1038/s41467-021-21143-1 (PMC7943601; doi:10.1038/s41467-021-21143-1)
Supplement: Supplementary file 5 — Description of Additional Supplementary Files [file 41467_2021_21143_MOESM5_ESM.docx]

**Description of additional supplementary information**

**Title: Supplementary Video 1**

**Description:** Video illustrating the conformational changes of the *S. enterica* sv. Typhimurium export apparatus M-gate during protein secretion. Surface representations of the export apparatus proteins SpaP_1-5_ (blue colors), SpaR (orange), SpaQ_1-4_ (yellow colors) and the SptP3x-GFP substrate (magenta) are shown in the closed and open states. Subsequently, M-gate SpaP_1-5_ Met185-7 and SpaR Phe212 residues are shown in surface representations. During substrate translocation, these methionines from each SpaP monomer shift open to allow passage of the substrate. The M-gate forms a hydrophobic, movable gasket around the substrate. Hydrophobicity coloring: green: hydrophilic; white: neutral; gold: hydrophobic.

**Title: Supplementary Video 2.**

**Description:** Video illustrating the conformational changes of the *S. enterica* sv. Typhimurium export apparatus SpaR lid during protein secretion. Surface representations of the export apparatus proteins SpaP_1-5_ (blue colors), SpaR (orange), SpaQ_1-4_ (yellow colors) and the SptP3x-GFP substrate (magenta) are shown and SpaR is later depicted in cartoon cylinder representation. In the closed EA state, the SpaR loop/lid rests on top of the M-gate and during substrate translocation the loop shifts upwards to open the channel. In the open confirmation, the loop assumes two separate states where an Ile114 (depicted in red) makes contact with the substrate backbone in state 1 or shifts away in state 2.
