# Supplementary material for: Substrate-engaged type III secretion system structures reveal gating mechanism for unfolded protein translocation
Source: Nat Commun. 2021 Mar 9;12:1546. doi: 10.1038/s41467-021-21143-1 (PMC7943601; doi:10.1038/s41467-021-21143-1)
Supplement: Supplementary file 6 — Reporting Summary [file 41467_2021_21143_MOESM6_ESM.pdf]

nature research

Corresponding author(s): Prof. Dr. Thomas Marlovits

Last updated by author(s): Dec 23, 2020

Reporting Summary

Nature Research wishes to improve the reproducibility of the work that we publish. This form provides structure for consistency and transparency in reporting. For further information on Nature Research policies, see our Editorial Policies and the Editorial Policy Checklist.

Statistics

For all statistical analyses, confirm that the following items are present in the figure legend, table legend, main text, or Methods section.

n/a	Confirmed	
☐	☒	The exact sample size (*n*) for each experimental group/condition, given as a discrete number and unit of measurement
☐	☒	A statement on whether measurements were taken from distinct samples or whether the same sample was measured repeatedly
☐	☒	The statistical test(s) used AND whether they are one- or two-sided<br>*Only common tests should be described solely by name; describe more complex techniques in the Methods section.*
☒	☐	A description of all covariates tested
☒	☐	A description of any assumptions or corrections, such as tests of normality and adjustment for multiple comparisons
☐	☒	A full description of the statistical parameters including central tendency (e.g. means) or other basic estimates (e.g. regression coefficient) AND variation (e.g. standard deviation) or associated estimates of uncertainty (e.g. confidence intervals)
☐	☒	For null hypothesis testing, the test statistic (e.g. *F*, *t*, *r*) with confidence intervals, effect sizes, degrees of freedom and *P* value noted<br>*Give P values as exact values whenever suitable.*
☒	☐	For Bayesian analysis, information on the choice of priors and Markov chain Monte Carlo settings
☒	☐	For hierarchical and complex designs, identification of the appropriate level for tests and full reporting of outcomes
☒	☐	Estimates of effect sizes (e.g. Cohen's *d*, Pearson's *r*), indicating how they were calculated

Our web collection on statistics for biologists contains articles on many of the points above.

Software and code

Policy information about availability of computer code

Data collection	CryoEM: Thermo Fisher Scientific, EPU 1 and 2. Negtaive stain EM: FEI/Thermo Fisher Scientific, Talos v.1.15.3 and TEM Imaging and analysis software v.5.0. Western blotting: Intas Science Imaging Instruments GmbH, ChemoStar Touch v. 0.5.65.
Data analysis	MotionCor2 v.1.2.1 and v.1.3, CTFind v.4.1.13, crYOLO v.1.2 and v.1.4, Relion 3.0 and Relion 3.1-beta, HOLE program v.2.2, Phenix v.1.18.2, Chimera v.1.13.1 and v.1.14, ChimeraX v.0.8 and v.0.93, MolProbity v.4.5.1., COOT v.0.8.9.1 and 0.9-beta, StarMap (v. 1.1.12), EMRinger v.1.00, SWISS-MODEL server, Phyre v.2, Rosetta v.3.1 and 3.12, ISOLDE v.1.0b5, Phenix.real_space_refine v.1.18-6831, Pymol v.2.40, Graphpad Prism v.8.4.3, PDBePISA v.1.52, ImageJ v.1.50i

For manuscripts utilizing custom algorithms or software that are central to the research but not yet described in published literature, software must be made available to editors and reviewers. We strongly encourage code deposition in a community repository (e.g. GitHub). See the Nature Research guidelines for submitting code & software for further information.

Data

Policy information about availability of data

All manuscripts must include a data availability statement. This statement should provide the following information, where applicable:

- Accession codes, unique identifiers, or web links for publicly available datasets
- A list of figures that have associated raw data
- A description of any restrictions on data availability

The data that support the findings of this study are available from the corresponding author upon request. CryoEM maps and atomic coordinates have been deposited at the PDB/EMDB, and will be immediately released upon publication. The accession codes are: EMD-11780, PDB ID 7AGX,EMD-11781, PDB ID 7AH9, 7AHI

Field-specific reporting

Please select the one below that is the best fit for your research. If you are not sure, read the appropriate sections before making your selection.

☒ Life sciences ☐ Behavioural & social sciences ☐ Ecological, evolutionary & environmental sciences

For a reference copy of the document with all sections, see nature.com/documents/nr-reporting-summary-flat.pdf

Life sciences study design

All studies must disclose on these points even when the disclosure is negative.

Sample size	For the substrate-engaged cryoEM data set, 14450 micrograph movies were collected and 837325 coordinates were picked. For the apo state cryoEM data set, 10433 micrograph movies were collected and 234102 coordinates were picked. The rationale for these sample sizes was simply to maximize the amount of micrographs and particles imaged during cryoEM data collection to achieve the highest resolution structure possible. The substrate density is weaker and therefore more micrographs were collected for this sample. Picking models were generated to maximize the number of particles picked from each micrograph.
Data exclusions	For the substrate-engaged cryoEM data set, 759914 coordinates were excluded during cryoEM data processing. For the apo state cryoEM data set, 179611 coordinates were excluded during cryoEM data processing. Particles were excluded based on their estimated resolution with classes corresponding to the highest-resolution particles being used for the final structure. Also, particle picking models were generated to maximize the number of picked particles, and therefore coordinates corresponding to aggregated proteins or contamination were excluded.
Replication	CryoEM data collection parameters are provided in Supplementary Table 1. Given amount of time and resources needed for large-scale CryoEM data collections and extensive imaging processing, further replicative data collections were not repeated. For the bacterial growth assays, 9 independent measurements were acquired from three independent experiments and were all successful. For needle complex counting experiments, osmotically shocked cells were visualized 3 times with similar results. Needle complex countings were repeated by three colleagues. Complexes were counted from 20 cells for each strain. For secretion assays and Western blotting, 3 independent experiments were conducted. All were successful.
Randomization	In single particle cryoEM, particles were split randomly into two groups and then processed to calculate Fourier-shell correlation coefficients following Gold standard methods.
Blinding	Blinding was used during the needle complex counting assay. Strain IDs were hidden from colleagues during counting.

Reporting for specific materials, systems and methods

We require information from authors about some types of materials, experimental systems and methods used in many studies. Here, indicate whether each material, system or method listed is relevant to your study. If you are not sure if a list item applies to your research, read the appropriate section before selecting a response.

Materials & experimental systems

n/a	Involved in the study
☐	☒ Antibodies
☒	☐ Eukaryotic cell lines
☒	☐ Palaeontology and archaeology
☒	☐ Animals and other organisms
☒	☐ Human research participants
☒	☐ Clinical data
☒	☐ Dual use research of concern

Methods

n/a	Involved in the study
☒	☐ ChIP-seq
☒	☐ Flow cytometry
☒	☐ MRI-based neuroimaging

Antibodies

Antibodies used	Rabbit anti-needle complex pAb (InvG, PrgH, PrgK, PrgJ and PrgI) generated in-house<br>Rabbit anti-SptP pAb generated in-house<br>Rabbit anti SipA pAb generated in-house<br>Mouse anti-Flag mAb (Merck/Sigma-Aldrich Product number F1804)
Validation	The antibodies have been used and validated in the previous publication: Radics, J., Königsmaier, L. & Marlovits, T. C. Structure of a pathogenic type 3 secretion system in action. Nat. Struct. Mol. Biol. 21, 82–87 (2014).
